# Role of a polyphenol-enriched preparation on chemoprevention of mammary carcinoma through cancer stem cells and inflammatory pathways modulation

**DOI:** 10.1186/s12967-016-0770-7

**Published:** 2016-01-14

**Authors:** Tri Vuong, Jean-François Mallet, Maria Ouzounova, Sam Rahbar, Hector Hernandez-Vargas, Zdenko Herceg, Chantal Matar

**Affiliations:** Nutritional Sciences Program, Faculty of Health Sciences, University of Ottawa, R2057 Roger Guindon Hall, 451 Smyth Road, Ottawa, ON K1H 8M5 Canada; Cellular and Molecular Medicine, Faculty of Medicine, University of Ottawa, Ottawa, Canada; Cancer Center, Georgia Regents University, Augusta, GA USA; International Agency for Research on Cancer, Lyon, France

**Keywords:** Polyphenols, Breast cancer stem cells, Tumor, Metastasis, STAT3, MAPKs

## Abstract

**Background:**

Naturally occurring polyphenolic compounds from fruits, particularly from blueberries, have been reported to be significantly involved in cancer chemoprevention and chemotherapy. Biotransformation of blueberry juice by *Serratia vaccinii* increases its polyphenolic content and endows it with anti-inflammatory properties.

**Methods:**

This study evaluated the effect of a polyphenol-enriched blueberry preparation (PEBP) and its non-fermented counterpart (NBJ), on mammary cancer stem cell (CSC) development in in vitro, in vivo and ex vivo settings. Effects of PEBP on cell proliferation, mobility, invasion, and mammosphere formation were measured in vitro in three cell lines: murine 4T1 and human MCF7 and MDA-MB-231. Ex vivo mammosphere formation, tumor growth and metastasis observations were carried out in a BALB/c mouse model.

**Results:**

Our research revealed that PEBP influence cellular signaling cascades of breast CSCs, regulating the activity of transcription factors and, consequently, inhibiting tumor growth in vivo by decreasing metastasis and controlling PI3K/AKT, MAPK/ERK, and STAT3 pathways, central nodes in CSC inflammatory signaling. PEBP significantly inhibited cell proliferation of 4T1, MCF-7 and MDA-MB-231. In all cell lines, PEBP reduced mammosphere formation, cell mobility and cell migration. In vivo, PEBP significantly reduced tumor development, inhibited the formation of ex vivo mammospheres, and significantly reduced lung metastasis.

**Conclusions:**

This study showed that polyphenol enrichment of a blueberry preparation by fermentation increases its chemopreventive potential by protecting mice against tumor development, inhibiting the formation of cancer stem cells and reducing lung metastasis. Thus, PEBP may represent a novel complementary alternative medicine therapy and a source for novel therapeutic agents against breast cancer.

## Background

Life-style changes significantly contribute to cancer prevention and are considered an important paradigm in translational medicine [1]. For example, a dietary intervention showed that a few months of following a Mediterranean diet are sufficient to favorably modify the metabolic/endocrine characteristics of breast cancer survivors [2]. In fact, breast cancer patients are among the highest users of integrative medicine in conjunction with conventional oncology care [3]. Currently, cancer preventive phytochemicals are receiving increasing attention regarding their impact on Cancer Stem Cell (CSC) self-renewal pathways [4]. In line with these reports, our preliminary results have shown that repression of breast CSCs by fermented blueberry preparation, named Polyphenols-Enriched Blueberry Preparation (referred hereafter as PEBP), supports diet-mediated targeting of CSCs. The chemopreventive effects of blueberry polyphenolics on breast cancer are well-known [5, 6]. For example, phenolic extracts from European blueberry were shown to inhibit proliferation and induce apoptosis in breast cancer cells [7]. Therefore, increasing the phenolic content of blueberry might enhance its anticancer properties and reduce its metastatic potential. Indeed, biotransformation of blueberry juice with a novel strain of bacteria isolated from the blueberry flora increases its phenolic content and antioxidant activity [8].

CSCs, a highly tumorigenic cell subtype, are emerging as key drivers of cancer [9, 10]. CSCs in breast cancer have been identified as CD44^+^/CD24^low^ phenotype and are able to grow as spheres named, in this case, mammospheres [11, 12]. Interleukin 6 (IL-6) and its major effector, the signal transducer and activator of transcription 3 (STAT3), are part of an important inflammation-associated pathway in malignancies, and are highly involved in CSC development and progression [13]. STAT3 has been recently recognized as a key therapeutic target to reduce tumor growth [14] and metastasis [15] in different types of cancer. The persistent self-renewal observed in CSCs was reported to be epigenetically controlled in the IL-6/STAT3/phosphatidylinositol 3-kinase (PI3K) signaling pathway [16]. STAT3 with PTEN is part of the positive feedback loop that underlies the epigenetic switch that links inflammation to cancer. Thus, prevention or inhibition of deregulation in the PI3K/STAT3/PTEN signaling pathway could be beneficial for the treatment and better outcome of breast cancer. Several signal transduction pathways, such as the extracellular-signal-regulated kinase/mitogen-activated protein kinase (Erk/MAP) pathway and PI3K pathway have been implicated in mammary carcinogenesis [17].

Moreover, members of the mitogen-activated protein kinase (MAPK) pathways have been well studied for their role in controlling cellular responses to the environment and in regulating gene expression, cellular growth and apoptosis in cancer [18, 19]. The extracellular signal-regulated kinases (ERKs)-1/2 were linked to cell proliferation and survival, whereas the stress-activated MAPKs, p38 and c-Jun N-terminal kinase (JNK), were connected to apoptosis [20]. Controlling MAPK pathways was shown to impact CSC-promoting IL-6 and modify CSC-like behavior [21].

Different studies have shown that the fermentation of PEBP greatly increased its antioxidant potential [8, 22] and endowed it with novel anti-inflammatory [23], antidiabetic [24, 25] and other biological activities [23]. Importantly, the anti-inflammatory effects of PEBP seemed to be connected to IL-6 related pathways, as demonstrated by decreasing hyperglycemia, activating AMPK pathways and mimicking Metformin metabolic effects [24]. Additionally, our studies have revealed that PEBP increases adiponectin secretion [24], probably by counteracting reactive oxygen species [26] and inhibiting the pro-inflammatory cytokines [27]; two mechanisms that contribute to the inflammatory response. Indeed, inflammation is linked to obesity, diabetes and cancer [28]. The goal of this study was to investigate the anticarcinogenic effects of polyphenol-enriched blueberry preparation (PEBP) on breast cancer stem cell development in cell models and in vivo, as well as to study the involvement of STAT3 and MAPKs signaling pathways in its chemopreventive activities.

## Methods

### Preparation of blueberry juices

Mature lowbush blueberries (*Vaccinium angustifolium* Ait.) were purchased from Cherryfield Foods Inc. (Cherryfield, ME) as fresh and untreated fruits. Blueberry juice was extracted by blending the fruit (100 g) in a Braun Type 4259 food processor. The fruit mixture was then centrifuged at 500×*g* for 10 min to remove insoluble particles. The resulting juice was sterilized using 0.22 µm Express Millipore filters (Millipore, Etobicoke, ON).

*Serratia vaccinii* bacteria were cultured as previously described [8]. Blueberry and polyphenol-enriched blueberry preparation have been partially characterized elsewhere [8, 29].

### Cell culture

Murine 4T1, a 6-Thioguanine resistant cell line, human MCF-7 and human MDA-MB-231 cell lines were obtained from American Type Cell Collection (ATCC; Chicago, IL, USA). ATCC authenticated the human cell lines by using short tandem repeat profiling and the mice cell line was confirmed to be from mice by cytochrome C oxidase 1 gene assay. MCF-7 cells were cultured in MEM, 4T1 and MDA-MB-231 in RPMI-1640, media containing FBS (10 %, v/v) (ATCC), penicillin (100 µU/ml), streptomycin (100 µg/ml) (Sigma-Aldrich, Oakville, ON) at 37 °C in a humidified atmosphere with 5 % CO_2_.

### Cell viability

Cell viability was assessed by water soluble tetrazolium salts (WST-1) and Lactate Dehydrogenase (LDH) assays (Roche, Laval, QC). After a 24 h treatment, supernatants were collected for LDH assay following the manufacturer’s instructions. The absorbance was measured with the μ-Quant plate reader (Bio-Tek, Winooski, VT) [30].

### Cell motility

Cells were plated in a six-well plate at density of 1 × 10^6^ cells/0.2 ml/well and allowed to form a confluent monolayer for 24 h. The monolayer was then scratched with a pipette tip, washed with RPMI-1640 to remove floating cells, and photographed (time 0). The cells were treated with NBJ or PEBP for 24 h. The cells were then photographed again at three randomly selected sites per well. Cell motility was expressed as a percent of the surface area covered by migrating cells compared with time 0 [30].

### Cell invasion

The cell invasion assay was performed on a polyethylene terephthalate (PET) membrane (8 µm pore size) in a Tissue Culture (TC) insert (BD biosciences, Mississauga, ON) according to the manufacturer’s instructions. In short, cells are incubated in the superior chamber for 24 h. The insert is then transferred to a new plate containing HBSS supplemented with 4 µg/ml of Calcein AM for 1 h. The intensity of the fluorescence is measured and is expressed as a ratio of the control well without treatment [31].

### Mammospheres formation

Adherent cells were detached by trypsin and single cells were counted using the Countess automated cell counter (Invitrogen, Burlington, ON). For tumor tissue, approximately 0.05 g of each tumor was minced and dissociated in RPMI-1640 media containing 300 U/ml collagenase (Sigma), and 100 U/ml hyaluronidase (Sigma) at 37 °C for 2 h. The cells were sieved sequentially through a 100 µm and a 40 µm cell strainer (BD Biosciences) to obtain a single cell suspension, and counted in a hemocytometer.

Single cells were plated in ultralow attachment 96-well plates (Costar) at 10^3^ cells/0.2 ml/well, in the presence/absence of PEBP and NBJ, in DMEM-F12 (Invitrogen), supplemented with 10 ng/ml EGF, 20 ng/ml bFGF, 5 µg/ml insulin, 1 mM sodium pyruvate, 0.5 µg/ml hydrocortisone, and penicillin/streptomycin (0.05 mg/ml) (Sigma) [16]. Cells grown in these conditions as non-adherent spherical clusters of cells or mammospheres were counted after 4–7 days.

### IL-6 determination

BD OptEIA Mouse IL-6 ELISA sets (BD Biosciences) were used to measure extracellular IL-6 production by mammospheres following the manufacturer’s instructions.

### Western blot analysis

Cells in mammospheres formation conditions were collected and lysed after 1, 2, 6 and 24 h treatment with/without PEBP and NBJ. Cell lysates were run on a 10 % acrylamide gel, transferred to a PVDF membrane, and probed with either anti-phosphorylated STAT3 (1:1000), PI3K (1:1000), Akt (1:1000), PTEN (1:1000), p38 MAPK (1:1000), ERK1/2 (1:1000), SAPK/JNK (1:1000), β-Actin (1:1000) (Cell Signaling Tech. Inc., Danvers, MA, USA). Bands were visualized via chemiluminescence using horseradish peroxidase-conjugated secondary antibodies. Bands were quantified using β-actin as loading control by Bio-Rad Quantity One software (Bio-Rad, Mississauga, ON).

### Animals

Six- to eight-week-old BALB/c female mice weighing 18–20 g (Charles River, Montreal, QC) were randomly distributed into seven experimental groups: control, NBJ 12.5 %, NBJ 25 %, NBJ 50 %, PEBP 12.5 %, PEBP 25 % and PEBP 50 %. Each experimental group consisted of 8 mice housed in a controlled atmosphere (temperature 22 ± 2 °C; humidity 55 ± 2 %) with a 12 h light/dark cycle. Mice were maintained and treated in accordance with the guidelines of the Canadian Council on Animal Care. The protocol (ME-289) was approved by the Animal Care Committee of University of Ottawa.

While mice in the control group received normal water, mice in NBJ- and PEBP-groups received either NBJ or PEBP, incorporated in their drinking water at three concentrations: 12.5, 25 and 50 % (v/v) respectively. After 2 weeks of treatment, all mice received a subcutaneous injection of 4T1 cells (1400 cells/0.1 ml/mice) into the abdominal mammary gland fat pad. Three weeks after the inoculation, tumors and lungs were collected and weighed [32]. Mice consumed an average of 2.9 ml of juice each day and both blueberry juices were well tolerated and did not affect mice body weight.

### Lung metastasis

Lungs were minced and dissociated in RPMI-1640 media containing 300 U/ml collagenase (Sigma), at 37 °C for 15 min. After filtration through a 40 µm cell strainer (BD Biosciences), cells were collected and suspended in RPMI-1640 containing 10 % FBS (ATCC), penicillin/streptomycin (0.05 mg/ml) and 60 μM 6-Thioguanine (Sigma). The cells were plated in 10-cm culture dishes (Corning) at 37 °C in a humidified atmosphere with 5 % CO_2_. After 14 days, the lung cells were fixed by methanol and stained with 0.03 % methylene blue solution. All blue colonies were counted, one colony representing one clonogenic metastatic cell [32].

### Statistical analysis

Statistical analysis of the data by ANOVA and Bonferroni’s post hoc tests were performed using GraphPad Prism software version 5.04 (San Diego, CA, USA). Statistical significance was set at p ≤0.05. Data are reported as mean ± SEM.

## Results

### Inhibition of breast cancer cell proliferation

**Fig. 1:**
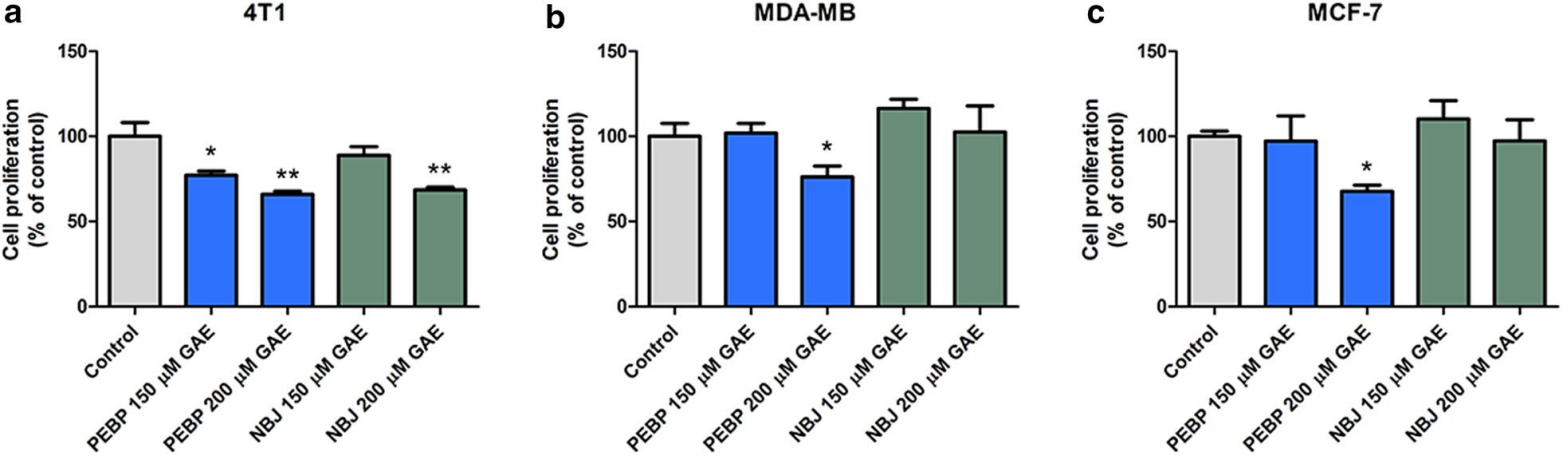
PEBP suppressed the growth of mammary carcinoma cell lines. Proliferation of 4T1 (**a**), MDAMB-231 (**b**), and MCF-7 (**c**) cells after treatment with either 150 or 200 μM GAE (gallic acid equivalent) of either polyphenol-enriched blueberry preparation (PEBP) or normal blueberry juice (NBJ) for 24 h. All values are means of 3 separated experiments ±SEM. *Denotes statistical significance at p ≤ 0.05 vs. control. **Denotes p ≤ 0.01 vs. control

At a concentration of 200 μM GAE, PEBP significantly inhibited the proliferation of 4T1, MDA-MB-231 and MCF-7 cancer cells by 34, 24 and 33 % respectively (Fig. 1), whereas the same concentration of NBJ only showed an inhibition of 32 % in 4T1 cell proliferation (Fig. 1a). No significant effects of NBJ were observed in MDA-MB-231 and MCF7 (Fig. 1b, c). Both PEBP and NBJ did not show any toxicity on the three cell lines at tested concentrations, as determined by an LDH assay (data not shown).

### Reduction of motility and invasiveness potential

**Fig. 2:**
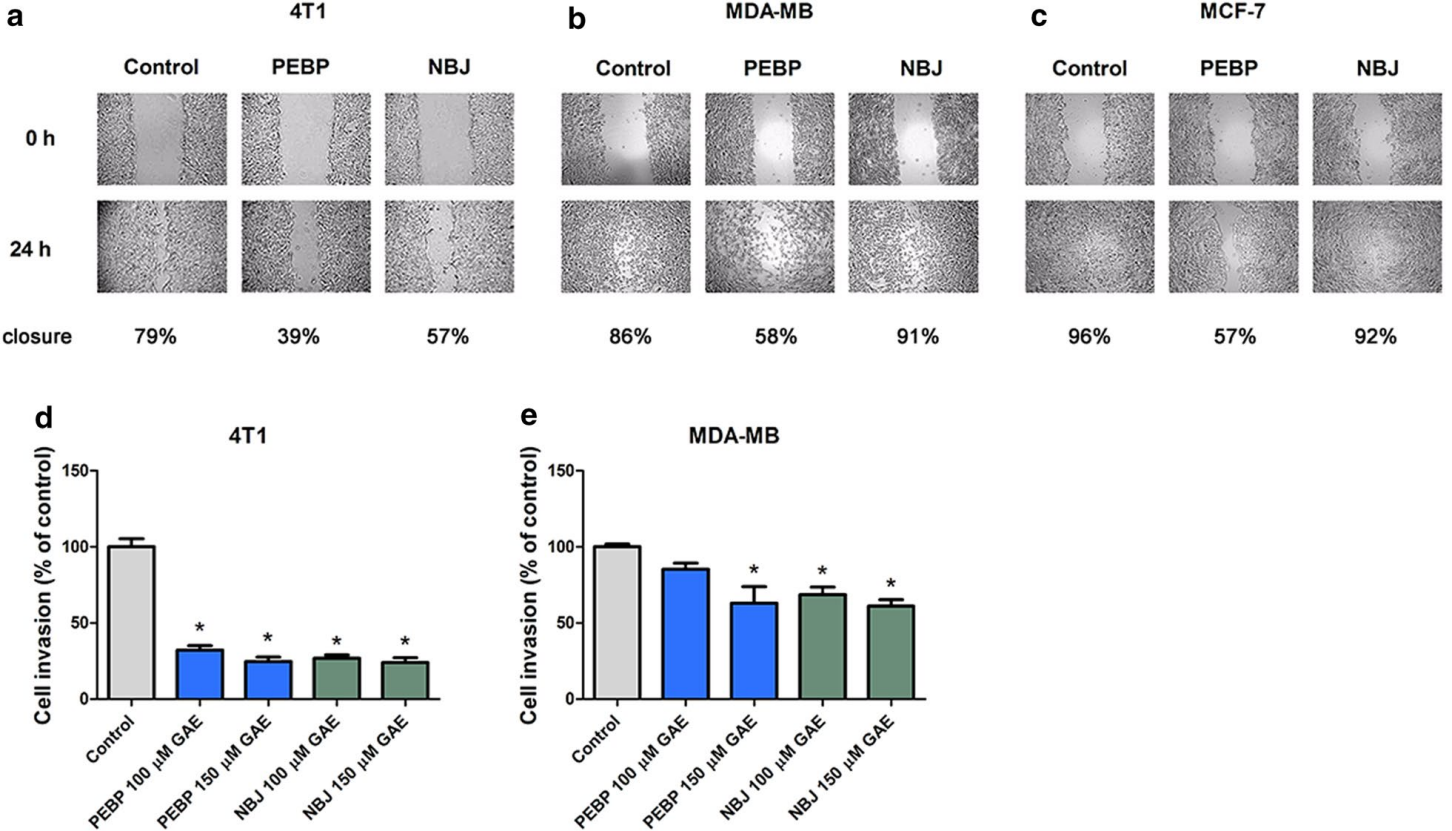
PEBP decreased motility and invasiveness in gel invasion experiment. Cell mobility of 4T1 (**a**), MDAMB-231 (**b**), and MCF-7 (**c**) cells after treatment with 100 μM GAE (gallic acid equivalent) of either polyphenol-enriched blueberry preparation (PEBP) or normal blueberry juice (NBJ) for 24 or 48 h and cell invasion of 4T1 (**d**) and MDAMB-231 (**e**) cells after treatment with either 100 or 150 μM GAE of PEBP or NBJ for 24 h. Contrast was enhance to better show cell motility. All values are means of 3 separated experiments ±SEM. *Denotes statistical significance at p ≤ 0.05 vs. control

Both NBJ and PEBP at 150 μM Gallic Acid Equivalent (GAE) significantly reduced the invasive ability of 4T1 and MDA-MB-231 (Fig. 2d, e). However, only PEBP exhibited an inhibitive effect on the motility of all three breast cancer cell lines (Fig. 2a–c). NBJ did not show any significant effect on cell motility as compared to the control.

### Inhibition of mammosphere formation

**Fig. 3:**
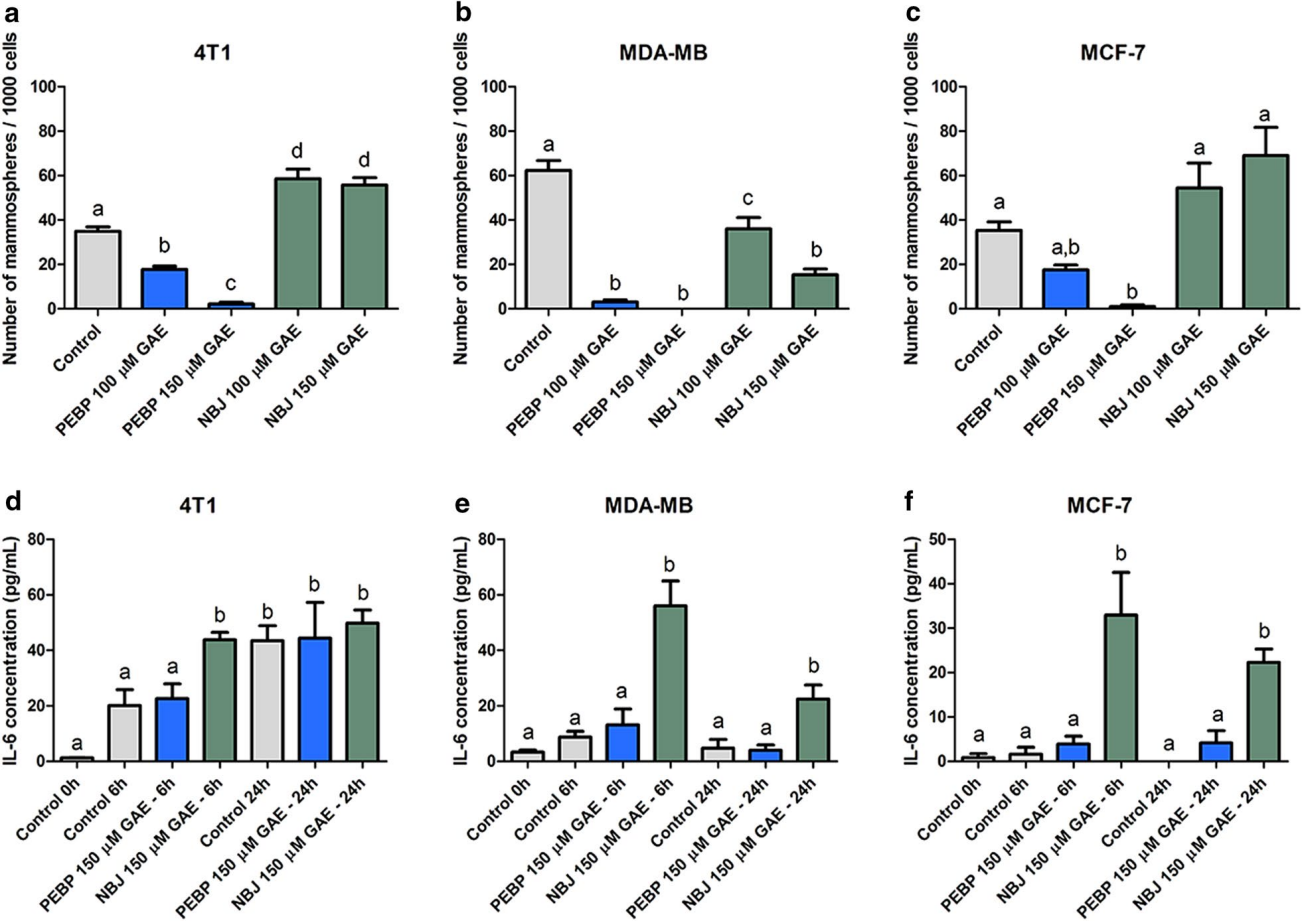
PEBP and NBJ decreased the formation of mammospheres in cell culture. Mammospheres formation of 4T1 (**a**), MDAMB-231 (**b**), and MCF-7 (**c**) cells after treatment with either 100 or 150 μM GAE (gallic acid equivalent) of either polyphenol-enriched blueberry preparation (PEBP) or normal blueberry juice (NBJ) for 4–7 days and IL-6 production by 4T1 (**d**), MDAMB-231 (**e**), and MCF-7 (**f**) cells after treatment with 150 μM GAE of PEBP or NBJ for 6 and 24 h. All values are means of 4 separated experiments ±SEM. *Bars* that have no letter in common are significantly different from each other (p ≤ 0.05)

PEBP significantly decreased the formation of mammospheres in all three cell lines (Fig. 3), and nearly total inhibition was observed at 150 μM GAE of PEBP. A treatment with the same concentration of NBJ only exhibited an inhibition of 75 % in MDA-MB-231 (Fig. 3b), whereas it significantly increased the formation of mammospheres in 4T1 by 60 % (Fig. 3a).

### Inhibition of IL-6/STAT3/PI3K signaling pathway

A 6 h-treatment with NBJ in mammosphere formation conditions significantly elevated the secretion of IL-6 in all three cell lines (Fig. 3d–f), while PEBP did not induce any modification as compared to the control cells.

**Fig. 4:**
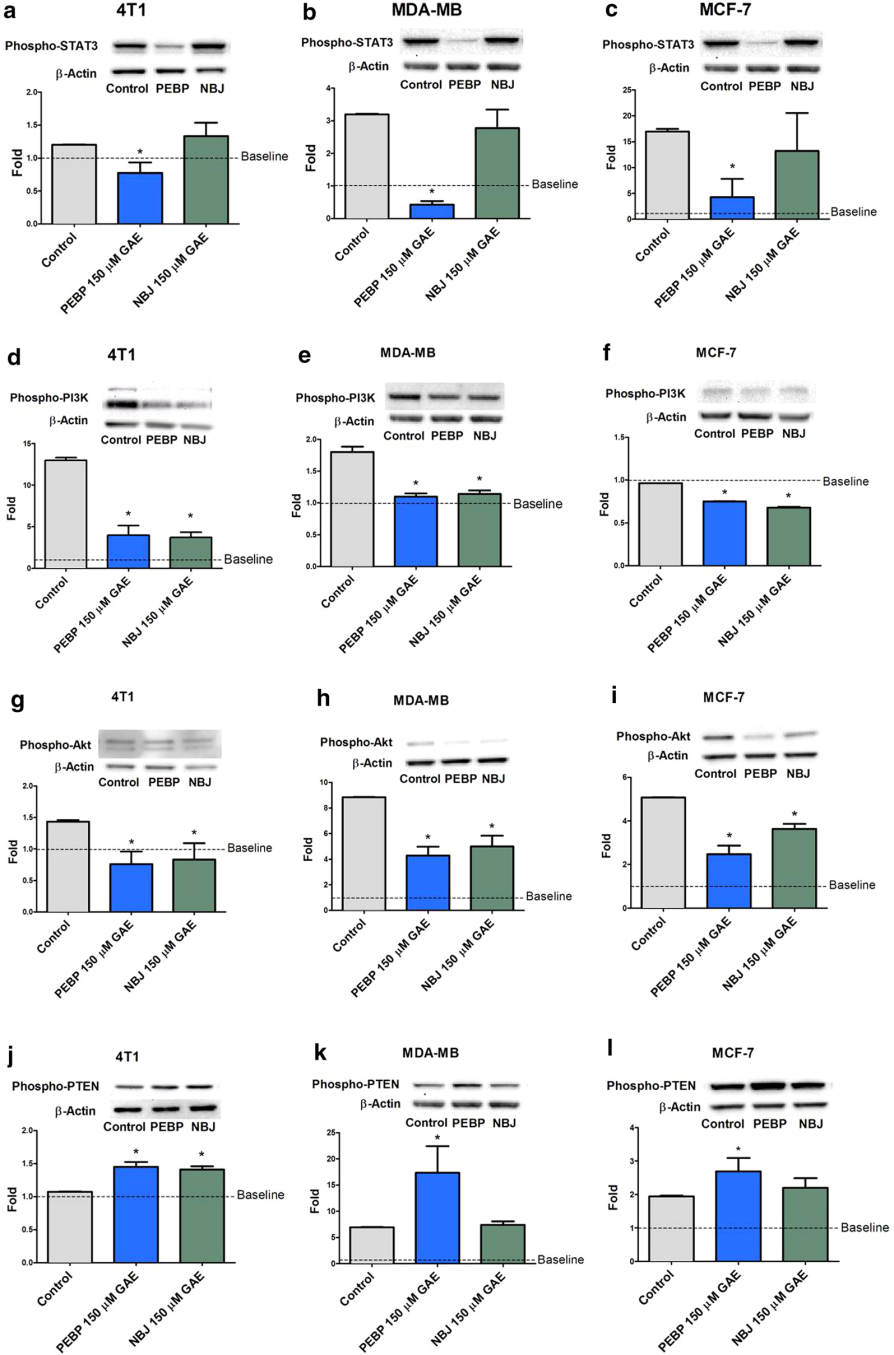
PEBP inhibited STAT3/PI3K/Akt signaling pathway. Phosphorylation of STAT3, PI3K, Akt, and PTEN in 4T1 (**a**, **d**, **g**, **j**), MDAMB-231 (**b**, **e**, **h**, **k**), and MCF-7 (**c**, **f**, **i**, **l**) mammospheres after treatment with 150 μM GAE (gallic acid equivalent) of either polyphenol-enriched blueberry preparation (PEBP) or normal blueberry juice (NBJ) for 6 h. All values are means of 3 separated experiments ±SEM. Baseline represent the level of phosphorylation present in cells not exposed to the mammospheres forming medium. *Denotes statistical significance at p ≤ 0.05 vs. control at 6 h

Moreover, PEBP significantly inhibited the phosphorylation of STAT3 and PI3K/Akt in all three cell lines. This inhibition started after a 6 h-treatment (Fig. 4a–i) and lasted up to 24 h (data not shown), whereas NBJ only decreased the phosphorylation of PI3K. Both PEBP and NBJ significantly enhanced the activity of PTEN in 4T1 (Fig. 4j), but only PEBP increased PTEN phosphorylation in MDA-MB-231 and MCF-7 (Fig. 4k–l).

### Alterations of MAPKs pathway

**Fig. 5:**
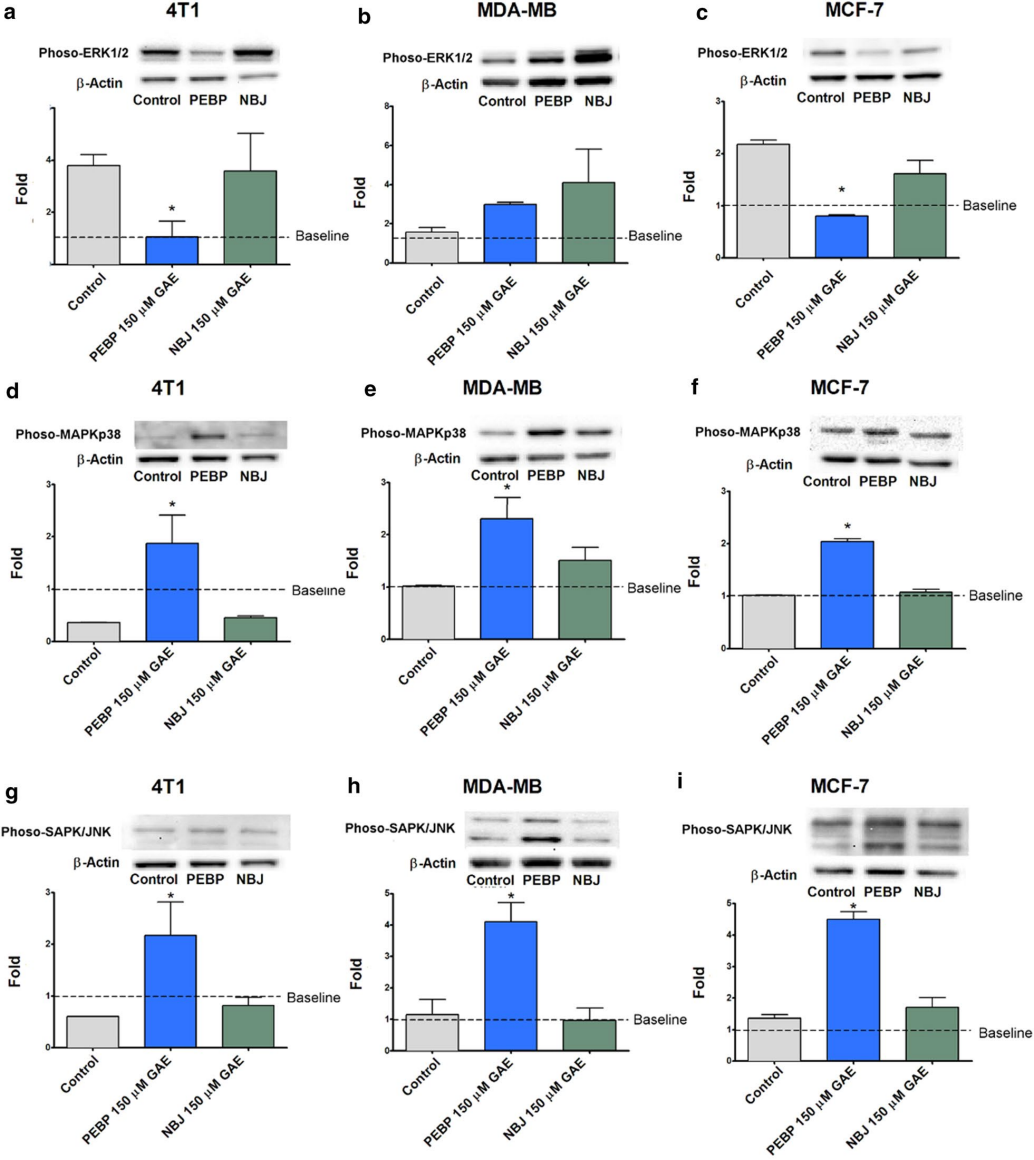
PEBP inhibited ERK1/2 but enhanced MAPKp38, and JNK signaling. Phosphorylation of ERK1/2, MAPK p38, and JNK in 4T1 (**a**, **d**, **g**), MDAMB-231 (**b**, **e**, **h**), and MCF-7 (**c**, **f**, **i**) mammospheres after treatment with 150 μM GAE (gallic acid equivalent) of either polyphenol-enriched blueberry preparation (PEBP) or normal blueberry juice (NBJ) for 2 h. All values are means of 3 separated experiments ±SEM. Baseline represent the level of phosphorylation present in cells not exposed to the mammospheres forming medium. * Denotes statistical significance at p ≤ 0.05 vs. control at 2 h

Starting from 1 h after the addition of PEBP, a significant inhibition of ERK1/2 phosphorylation was observed in 4T1 and MCF-7 (Fig. 5a, c). PEBP also increased MAPK p38 and JNK/SAPK phosphorylation in all three cell lines. Their inhibited- or activated-state attainted the maximal level after 2 h of treatment and remained stable up to 24 h (Fig. 5d–i). NBJ did not show any significant modification of the three MAPKs family members.

### Reduction of tumor growth, mammosphere formation and metastasis in vivo

**Fig. 6:**
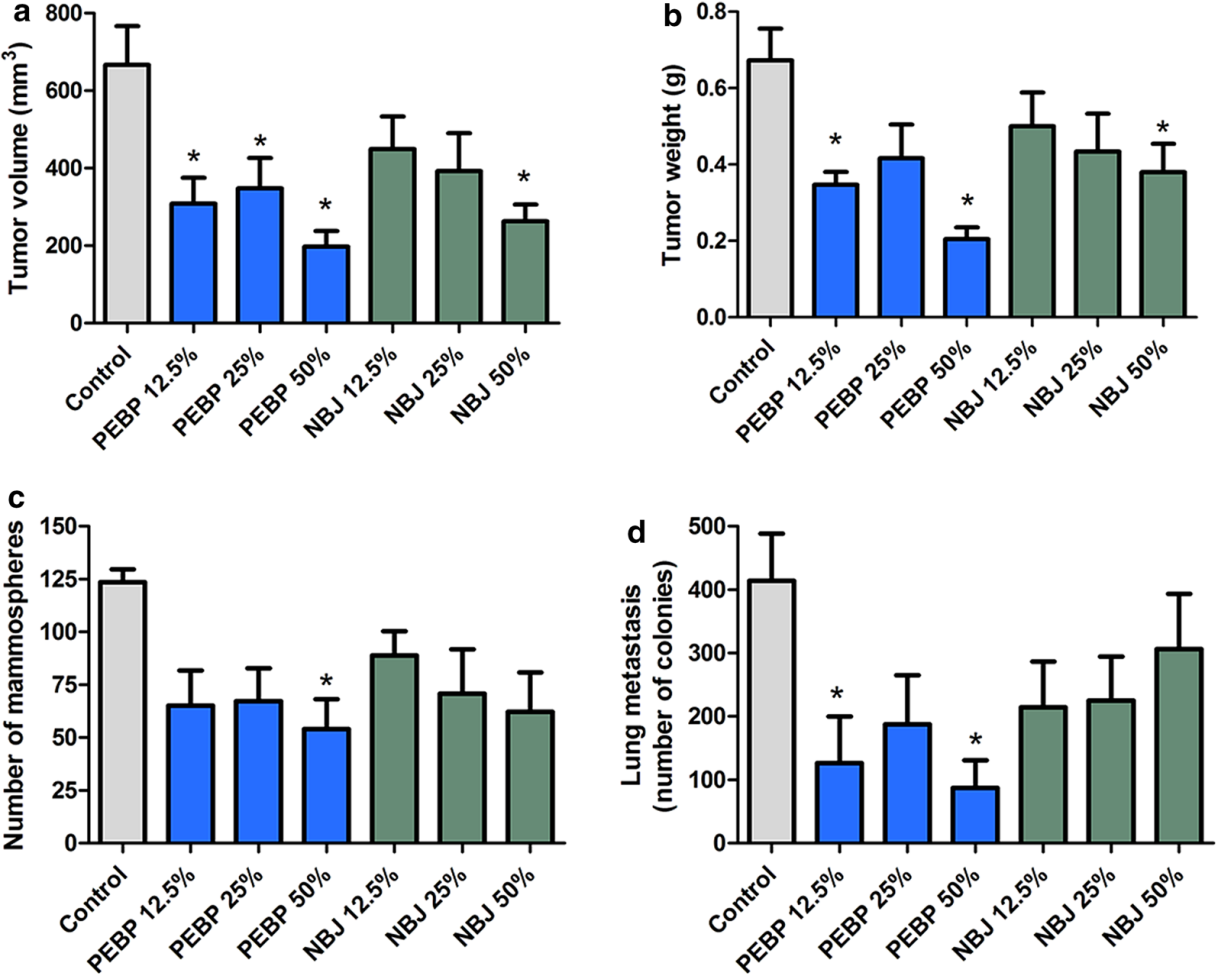
Antitumoral effects of PEBP in BALB/c mice model with 4T1 cell challenge. Tumor volume (**a**), tumor weight (**b**), mammospheres formation from primary tumor cells (**c**) and metastasis present in lungs (**d**) of mice that received a 2-week pre-treatment and a 3-week post-inoculation treatment with either polyphenol-enriched blueberry preparation (PEBP) or normal blueberry juice (NBJ) incorporated in drinking water at concentrations of 12.5 % (NBJ 12.5 % and PEBP 12.5 %), 25 % (NBJ 25 % and PEBP 25 %), and 50 % (NBJ 50 % and PEBP 50 %). All values are means of 2 separated experiments ±SEM (n = 16). *Denotes statistical significance at p ≤ 0.05 vs. control

As illustrated in Fig. 6, when administered chronically over a 5-week period, NBJ reduced tumor volume and weight in a dose-dependent manner. However, significant effects were only observed in the NBJ 50 % group, whereas all three doses of PEBP-treated mice displayed significant delays of tumor growth (Fig. 6a, b). Moreover, the mammosphere formation from tumoral primary cells was significantly reduced exclusively in tumors of PEBP 50 %-treated animals (Fig. 6c). Similarly, the treatment with PEBP significantly reduced the metastasis in lungs of PEBP-treated mice, while all of the other groups did not show a significant difference as compared to control animals (Fig. 6d).

## Discussion

Chemoprevention is an important part of integrative and translational medicine in oncology. Naturally occurring compounds, such as polyphenols in fruits, are increasingly recognized for their effects in controlling aberrant signaling pathways and inflammatory signals in CSCs. Our group has discovered that the fermented, probiotic-like product PEBP greatly accentuates its antioxidant potential and endows it with novel anti-inflammatory [22], antidiabetic [24, 25] and neuroprotective [23] biological properties. The common mechanisms underlining the multiple beneficial effects of PEPB are probably related to its capability to modulate the activity of global regulators that are associated with cellular transformation and inflammation. In addition, biotransformations involving fermentation and catabolic breakdown have been suggested to enhance bioavailability [33].

In fact, PEBP was found to inhibit adipogenesis and increase glucose uptake in muscle cells and adipocytes [25] through the activation of the AMP-activated kinase, mimicking Metformin activities [34, 35]. Particularly, the anti-inflammatory effect of PEBP is pointing out to the blockade of the STAT3 pathways (essential in CSCs, and inflammation) and the activation of AMPK, which in turn inhibits MAPK downstream (essential in diabetes and cancer).

In addition, PEBP mimics Metformin anti-inflammatory/antitumoral activities by inactivation of PI3K/AKT pathways. Metformin is now proposed as a major adjunct therapy in cancer with a powerful inhibitory effect on CSCs [36, 37]. This observation led us to further investigate the effect of PEBP on CSCs.

The antiproliferative effect of PEBP was observed in all three breast cancer cell lines at 200 μM GAE, whereas NBJ, at the same concentration, only had an effect in 4T1. NBJ did not show any antiproliferative effect in MDA-MB-231 as previously reported [38, 39]. This might be due to the low tested-doses in our study. Moreover, PEBP significantly inhibited the motility of all three cancer cell lines, which prompted further investigation for its antimetastatic activity in vivo. As expected, PEBP significantly reduced metastasis potential to the lung when tested in a murine breast cancer model.

There is now substantial evidence that many cancers, including breast cancer, are driven by a cellular subpopulation, identified as cancer stem cells, which mediate tumor metastasis and resistance to conventional therapies. Therefore, controlling CSC growth in breast cancer is a possible avenue to prevent tumor development and metastasis. Thus, the investigation of PEBP-induced molecular mechanisms that mediate CSC growth was important to clarify its anticancer and anti-metastatic activities. Indeed, our data indicated that PEBP significantly inhibited mammosphere formation in vitro. Moreover, its inhibitory effect was further confirmed by the reduction of ex vivo mammosphere development from PEBP-treated animals.

Polyphenols naturally have multi-target actions/mechanisms, which explain their wide spectrum of biological activities [40]. Their anti-inflammatory property is the key factor in the interface between inflammation and neoplasia [41]. At the cross road of cancer and inflammation, the STAT3 and MAPK pathways have been reported as crucial for CSC growth and their acquired EMT characteristics during metastasis [13, 42]. Depending on the cell type, the IL-6/STAT3-dependent pathways, such as the JAK/STAT [13], PI3K/AKT/NF-κB [43], or p38 MAPK [44], can enhance tumor growth and refractoriness to chemotherapy [13]. Therefore, our studies were conducted to examine the involvement of these pathways in PEBP’s antitumor activities. We demonstrated that IL-6 production, as well as STAT3 and PI3K phosphorylation, were decreased in CSC culture after PEBP treatment, when compared to the non-fermented control. Although, polyphenols from blueberry have demonstrated inhibitory activities on cancer cells via the control of inflammatory cytokines such as IL-6 [5], dramatic and biphasic increase of IL-6 occurs early in CSC cellular transformation [45], independently of STAT3 decrease. STAT3 signaling, an important inflammation-associated pathway in malignancies, has been recognized as a key therapeutic target to reduce tumor growth and metastasis [15]. Several signal transduction pathways such as STAT3, PI3K/AKT/NF-κB cascade, p38/MAPK/ERK, or the AMPK pathways play an important role in inflammation-mediated response at all stages of cancer development and refractoriness to chemotherapy [46]. Moreover, downstream effectors of the PI3K pathway include Akt, which is overexpressed in many cancer types and is associated with increased tumorigenicity [47, 48]. Our preliminary results showed that PEBP delayed the formation of CSCs in different types of cell culture and in vivo, through modulation of IL-6/STAT3, the PTEN/PI3K/AKT axis, and ERK/p38 in MAPK signaling pathways, which are all central nodes in CSC signaling and homeostasis [49] (Figs. 3, 5). We have demonstrated that STAT3, AKT, and PI3K are decreased, PTEN (a tumor suppressor gene upregulated by p53) is increased in a non-cell type dependent manner, and ERK1/2 was significantly inhibited in 4T1 and MCF7 (Fig. 5). In MAPK pathways, ERK1/2 is the most relevant to breast cancer. Increased expression of ERK1/2 was recently reported as driving endocrine resistance and breast cancer progression in an obesity-associated experimental model [50]. In fact, both PEBP and NBJ inhibited the phosphorylation of PI3K. These findings are consistent with previous reports, which attributed the inhibition of PI3K activity to the anticancer effects of blueberry [6, 38]. In our study, PEBP and NBJ also enhanced the activity of PTEN, an upstream inhibitor protein of PI3K, possibly via the inhibition of miRNA-21 expression [51]. These alterations, unfound with NBJ, could be exerted by the novel compounds that were produced during biotransformation and acted in concert on different types of receptors.

Treatment with PEBP rapidly increased p38-MAPK- and JNK- phosphorylation, which significantly reached its highest level at 2 h, and remained elevated for up to 24 h. PEBP reduced ERK1/2 phosphorylation in the same kinetic and cell-type independent manner. Modifications in MAPK family enzymes might contribute to the abolition of stem cell growth afforded by PEBP. Indeed, prolonged activations of JNK and MAPKp38 and/or inhibition of ERK1/2 induced apoptosis in most cancer cell lines [52–55]. The mechanisms by which PEBP modified MAPKs’ activities are unknown. In addition, PEBP-induced alterations of upstream MAPK members might inhibit the downstream STAT3/PI3K/Akt signaling, indicating an extensive cross-talk and interplay between the MAPK cascade and STAT3 pathways.

We further confirmed the in vivo anticancer and antimetastatic potential of PEBP using the 4T1-induced breast cancer model in BALB/c mice. The 4T1 tumor is highly tumorigenic and invasive and, unlike most tumor models, can spontaneously metastasize from the primary tumor in the mammary gland to multiple distant sites [56, 57].

Chronic administration of PEBP via incorporation in drinking water significantly reduced tumor volume and breast cancer stem cell development derived from the tumor. This diminution supports the low count of metastasis in lungs of PEBP-treated animals. Especially, PEBP anticancer and antimetastatic effects were observed at a therapeutic dose as low as 12.5 %, which, according to dose translation from animal to human using body surface area, corresponds to 1.2 cups of juice per day for humans [58]. In contrast, NBJ at the same dose did not show any significant effect. NBJ could show a decrease in tumor size and weight only at the dose of 50 %, which represents a substantial consumption of blueberry juice for humans. These results are consistent with findings from previous studies, which reported that feeding mice with blueberry extracts or whole fruit powder has an impact on inflammation and could delay tumor growth [6, 38, 59]. However, NBJ failed to achieve the reduction of breast cancer stem cells and metastasis observed with PEBP. Nonetheless, the process of preparing PEBP, which greatly increases the content in total phenolic compounds, could clarify its effectiveness at a low therapeutic dose as compared to NBJ. Furthermore, the novel antimetastatic potential of PEBP could be explained by the change of phenolic composition from NBJ to PEBP during the biotransformation process. Indeed, the biotransformation of blueberry juice not only increases its phenolic content, but also produces novel compounds [8]. One interesting possibility is that these novel compounds may possess more potent anticancer and antimetastatic properties that could have contributed to the observed reduction in tumor size and metastasis, as opposed to components of NBJ. In addition, the biotransformation process has probably broken down long polyphenol chains, which are poorly absorbed into gastro-intestinal tracts, increasing their bioavailability, and rendering PEBP highly functional [60].

## Conclusion

The results of the present study demonstrate that polyphenol-enriched blueberry preparation potently reduced the tumor growth and metastasis in mice. We have demonstrated that repression of breast Cancer Stem Cells (CSCs) by fermented blueberry supports a diet-mediated targeting of CSCs. We have provided evidence that PEBP selectively inhibits the inflammatory signature in CSCs through signaling pathways linked to the maintenance stemness and metastasis. The mechanisms of action involve, at least in part, alterations in the MAPKs cascade and inhibition of the STAT3 signaling pathway, involved in inflammatory pathways. The results convincingly demonstrated that PEBP, indeed, holds great promise as a chemopreventive agent and may represent a novel complementary therapy against breast cancer and metastasis. Conclusively, the prospective modulation of CSCs by nutrition will probably mark a major advance in preventing breast cancer and further optimizing the management of this significant disease. It is an important approach in translational medicine for specific integrative therapies that can be recommended as evidence-based supportive care for cancer patients.

## Abbreviations

CSC: cancer stem cells
ERK: extracellular signal-regulated kinase
IL-6: interleukine 6
JNK: c-Jun N-terminal kinase
GAE: gallic acid equivalent
MAPK: mitogen-activated protein kinase
NBJ: normal blueberry juice
PEBP: polyphenol-enriched blueberry preparation
STAT3: signal transducer and activator of transcription 3
